# Management of Septated Malignant Pleural Effusions

**DOI:** 10.1007/s13665-018-0194-3

**Published:** 2018-01-20

**Authors:** Radhika Banka, Dayle Terrington, Eleanor K. Mishra

**Affiliations:** 1 Norfolk and Norwich University Hospital NHS Foundation Trust, Norwich, Norfolk, UK; 2 University of East Anglia, Norwich, Norfolk, UK

**Keywords:** Pleural effusion, Septation, Fibrinolytic, Cancer

## Abstract

**Purpose of Review:**

We review recent studies of patients with septated malignant pleural effusions, to understand what the clinical implications for patients are and what evidence-based methods should be used to manage these effusions.

**Recent Findings:**

Fibrinolytics improve effusion size assessed radiologically in patients with a chest drain inserted for septated malignant pleural effusions but this does not translate into an improvement in breathlessness relief or pleurodesis success. Fibrinolytics have also been used in patients with septated effusions associated with indwelling pleural catheters, but dyspnoea relief has not been assessed in this population. Patients with septated effusions or extensive adhesions appear to have a worse prognosis.

**Summary:**

Patients with septated malignant pleural effusions have a poor prognosis and do not gain clinical benefit from fibrinolytics via chest drain. The role of fibrinolytics for septated effusions associated with indwelling pleural catheters requires further study.

## Introduction

Malignant pleural effusions (MPEs), defined as the presence of fluid in the pleural space secondary to cancer, are common and cause disabling dyspnoea. Cost of hospitalisations related to MPE was estimated to be over $5 billion in 2012 in the USA alone [1]. MPEs are caused by mesothelioma or metastatic cancer to the pleura. Patients have a median survival of several months [2]. Treatment aims to provide prolonged dyspnoea relief while minimising the number of pleural drainage procedures. This is achieved by either chest drainage and injection of an irritant, such as talc, or via an indwelling pleural catheter (IPC), a tunnelled semi-permanent catheter that is drained at home [3]. However, septations can develop within the fluid. These are formed from fibrin and divide the fluid into separate pockets. This leads to failure of complete drainage, leaving the patient with intractable dyspnoea. Another complication associated with MPEs is the development of trapped lung, in which chronically collapsed lung fails to reexpand following pleural fluid drainage, due to the development of a thickened visceral pleura. This article reviews our current understanding of how we should manage patients with septated MPEs.

## Aetiology

Development of pleural fluid septations is thought to be due to activation of the coagulation cascade, resulting in deposition of fibrin to form fibrin sheets. Chung et al. demonstrated that repeated thoracenteses over 3 days led to an increase in the levels of pleural fluid inflammatory markers (tumour necrosis factor (TNF)-alpha, interleukin (IL)-8 and neutrophil count) and activation of the coagulation cascade in the pleural space, as measured by plasminogen activator inhibitor type 1 (PAI-1) levels [4]. By day 6, 11/26 (42%) had developed septations visible on ultrasound. The patients who developed septations had greater increase in PAI-1 levels, supporting the hypothesis that activation of the coagulation cascade in pleural fluid leads to fibrin deposition and septation formation and suggests that one stimulus for this is invasive pleural procedures.

We compared patient characteristics of patients whose pleural effusion did not drain following chest drain insertion (presumed to be due to the presence of septations, recruited to a randomised trial of intrapleural fibrinolytics versus placebo for MPEs [5••]) and unselected patients with MPEs (recruited to a randomised trial of chest drain and pleurodesis versus IPC [2]), in order to identify patient characteristics associated with non-draining MPE [6•]. Patients with non-draining MPEs had a significantly higher pleural fluid lactate dehydrogenase (LDH) level (mean 1900 (SD 3100) versus 660 (SD 840), *p* < 0.001) and systemic C-reactive protein (CRP) levels (mean 117 (SD 80) versus 62 (SD 55), *p* < 0.001). These patients were on average 4 years older, but there was no difference in sex, performance status, type of malignancy or pleural fluid pH, glucose or total protein levels. This data suggests that patients with septated effusions have higher levels of systemic inflammation and pleural fluid metabolic activity. These variables may lead to activation of the coagulation cascade in the pleural space with fibrin deposition.

As well as thin fibrin septations, dense vascularised adhesions can be found in the pleural space. The relationship between adhesions and septations is not clear, although it seems a reasonable hypothesis that, with time, fibrinous septations become infiltrated with fibroblasts, and there is deposition of collagen, leading to development of adhesions. However, these terms are not clearly defined and use varies between authors.

## Incidence

The proportion of MPEs with septations is not known. Bielsa et al. found significant adhesions (defined as obstruction of one third or more of the vision on thoracoscopy) in 40% of patients [7]. Of the 692 patients with chest drain inserted for MPE screened for recruitment into a randomised controlled trial of intrapleural fibrinolytic versus placebo (TIME3), 115 did not drain fully, suggesting that about 16% of patients with MPE have sufficient septations to affect drainage [5••]. However, other factors may affect drainage completeness, such as trapped lung and negative pleural pressure. It is likely that mildly septated effusions still drain well because the septations do not form complete barriers to flow of fluid. However, heavy septations or dense adhesions may lead to failure to drain.

## Investigations

Septations are clearly visible on thoracic ultrasound. The increase in availability of ultrasound machines and of physician-performed thoracic ultrasound has led to an increase in the rate of recognition of pleural septations. Ultrasound prior to medical thoracoscopy has been shown to reliably identify adhesions and help improve pleural space access [8]. Our clinical practice has previously been to subjectively categorise effusions as unseptated, mildly septated, moderately septated and heavily septated. However, we recognised that this subjective assessment may not be reproducible between operators and centres. For this reason, we developed and validated an objective thoracic ultrasound septation score. This score categorises effusions based on the extent of septations (unseptated, heterogeneously septated and homogenously septated) and the number of septations at the most septated point (0, 1–2, 3–4, > = 5). This score shows good interrater reliability and corresponds to our previous subjective categorisation (personal data). In patients with chest drain inserted for a pleural effusion, we found a negative correlation between number of septations and dyspnoea relief [9].

Computer tomography is routinely performed in patients with suspected MPE. Septations are not visible on CT, although there may be signs to suggest that there are septations such as air pockets within the fluid or there may be visible adhesions between the visceral and parietal pleura.

Septations may also be seen at thoracoscopy and this allows the thoracoscopist to distinguish between thin fibrinous septations, which can be easily divided with the thoracoscope, and dense adhesions in which blood vessels are visible.

## Treatment

In patients with MPE who have developed a septated effusion, fibrinolytics have been used to dissolve the septations, with the ultimate aims of relieving breathlessness and improving pleurodesis success. Thoracic ultrasound performed before and after administration of pleural fibrinolytic demonstrates the dissolution of septations [10]. To date, there have been three randomised controlled trials of pleural fibrinolytics in patients with MPEs which have been treated with chest drain insertion. TIME3 randomised 71 patients with non-draining MPEs to either pleural urokinase (100,000 IU, three doses at 12-h interval) or placebo [5••]. Okur et al. randomised 47 unselected patients with MPE to either pleural streptokinase (250,000 IU, three doses at 12-interval) or no treatment [11]. Saydam el al. randomised 40 patients with multiloculated MPE to either pleural streptokinase (250,000 IU, four doses at 12-h interval) or saline [12].

In TIME3, we found patients had significant residual dyspnoea following urokinase/placebo administration, with no significant difference between the groups (mean VAS dyspnoea over 28 days 39 mm urokinase group versus 35 mm placebo group, *p* = 0.36). There was no difference in the proportion of patients who experienced a clinically significant decrease in dyspnoea (> = 19 mm, 13/36 urokinase group, 15/35 placebo group). Dyspnoea was not directly assessed in the trials by Okur and Saydam. Saydam et al. used oxygen use as a substitute for dyspnoea and found that 18 patients (90%) no longer required oxygen in the fibrinolytic group and 11 patients (55%) in the control group (*p* = 0.03). However, use of oxygen was determined by the treating physicians and the blinding is not clear, so this outcome may have been subject to bias or a placebo effect on patients.

All three studies showed an improvement in radiological appearance following pleural fibrinolytics, based on chest X-ray or computer tomography appearance. Although there was a trend to increased pleural fluid drainage in TIME3 and the study by Okur, this only reached statistical significance in the study by Saydam. These data demonstrate that pleural fibrinolytics decrease size of effusion, probably by increased pleural fluid drainage, but this does not translate into an improvement in dyspnoea. The implication for future trials is that direct measurement of dyspnoea should be the primary outcome.

Concerns have been raised that the use of pleural fibrinolytics in patients with MPE could reduce pleurodesis efficacy, but there were no significant differences in pleurodesis failure rates between patients who received pleural fibrinolytics and those who did not in the three trials discussed [5••, 11, 12]. It is likely that the short half time of pleural fibrinolytics in vivo means there is no impact on subsequent instillation of pleurodesis agents.

Septated effusions also develop in patients with IPCs, possibly due to the inflammatory effect of repeated drainage. This is estimated to occur in 6–14% of patients [2, 13]. Two retrospective case series of the use of pleural fibrinolytics in patients with IPCs who have developed symptomatic loculations have been published, one of 97 patients in a single centre where tissue plasminogen activator (tPA) was used [14] and the other from 4 IPC centres in 66 patients [15•], where a variety of fibrinolytics were used (tPA, 52 patients; urokinase, 12 patients; streptokinase, 2 patients). In the second series, the majority of fibrinolytic instillations were performed as outpatients (61%), and most patients (69.7%) only had a single dose of fibrinolytic. Pleural fluid drainage increased in 93% of patients and dyspnoea improved in 83% of patients.

The key concern over administration of pleural fibrinolytics in patients with pleural effusions is the risk of pleural haemorrhage. This is a particular concern in MPEs, where pleural tumour stimulates the development of new blood vessels, which can be friable and prone to bleeding. No cases of pleural or systemic haemorrhage were reported in the 80 patients receiving pleural fibrinolytics in the three trials discussed above [11, 12]. Of the four published case series of pleural fibrinolytics given via chest drain in MPE with a total of 101 patients, there were no cases of pleural haemorrhage reported [16–19]. In the two case series of patients receiving pleural fibrinolytics, a total of four cases of nonfatal pleural bleed out of 163 patients (2%) were reported [14, 15•]. This suggests that pleural fibrinolytics are safe in patients with MPEs, with a possible excess risk in patients with IPCs, but numbers are small and may be subject to reporting bias.

These data suggest that although pleural fibrinolytics given via chest drain to patients with septated MPEs are safe and can improve drainage volumes and effusion size, this does not translate to an improvement in dyspnoea. Given the poor prognosis and intractable dyspnoea in this patient group, we should question whether these patients should have a chest drain at all. Alternative palliative measures for controlling dyspnoea, such as intermittent therapeutic aspiration or opiates, may be more appropriate. The role of pleural fibrinolytics in patients with IPCs requires further study.

## Survival

MPEs are associated with a median survival of about 6 months [2]. However, there is some evidence that patients with pleural fluid septations or adhesions have a worse prognosis. We found patients with a septated, non-draining MPE to have a poor survival with a median time to death of 58 days (interquartile range 27–123) compared to 187 days (interquartile range 48–358) in unselected patients with MPE [2, 5•]. Bielsa et al. also found that patients with adhesions observed at thoracoscopy had a shorter survival, with patients with most extensive adhesions having a median time to death of 5 months compared to 9 months in patients with minimal or no adhesions [7]. This poor survival should be taken into account when managing these patients.

## Parallels with Pleural Infection

Pleural infection, with both typical bacteria and tuberculosis, is also associated with septation development. This makes the effusion more difficult to drain. As with MPEs, randomised trials have demonstrated that fibrinolytics alone improve pleural fluid drainage and radiological appearance but this does not translate into improved clinical outcomes (mortality and need for surgery) [20]. Subsequent work has suggested that the use of DNAse in conjunction with fibrinolytics may reduce need for surgery and length of hospital stay [21]. This work has been applied to patients with MPEs, with a published case series of use of DNAse/alteplase in patients with MPEs [19]. However, it should be noted that this is a different patient population with a different pathology with a different outcome of choice (relief of dyspnoea versus resolution of sepsis).

## Future Directions

There is a role for a longitudinal cohort study to understand why septated effusions develop, their incidence and clinical significance. This would help us understand whether fibrous septations progress into vascularised adhesions or whether these are separate processes. This will help improve treatment algorithms, for example, demonstration that repeated pleural procedures increase risk of septation development will strength the argument for going straight to a definitive treatment. Clinical trials of DNAse/fibrinolytics in septated effusions and fibrinolytics in non-draining IPCs will guide future management but dyspnoea relief must be the primary outcome, rather than radiological appearance or volume drained. Identification of trapped lung should also be an outcome measure. However, given that 692 patients were screen to randomise 71 in our trial of non-draining MPEs, it is unlikely that further adequately-powered trials will be performed in this population.

## Summary

Septated MPEs are associated with poor prognosis. Although fibrinolytics appear to improve effusion size in patients with chest drains, this does not translate into relief of breathlessness, and alternative palliative measures should be used in these patients. The role of fibrinolytics in patients with IPCs needs further study.
